# A German version of the Oral Impacts of Daily Performances—reliability and validity

**DOI:** 10.1007/s00784-023-05437-w

**Published:** 2024-01-04

**Authors:** Anna-Luisa Klotz, Dennis Prager, Peter Rammelsberg, Alexander Jochen Hassel, Andreas Zenthöfer

**Affiliations:** 1 Dental School, Department of Prosthodontics, University of Heidelberg, Im Neuenheimer Feld 400, 69120 Heidelberg, Germany; 2 Private Practice Dr. Prager, 69120 Heidelberg, Germany

**Keywords:** Oral health–related quality of life, Reliability, Validity, Assessment tool, dPROM

## Abstract

**Objective:**

The Oral Impact of Daily Performances (OIDP) is a dental patient–reported outcome measure (dPROM) for the estimation of oral health–related quality of life (OHRQoL) and takes the frequency as well as the severity of problems into account; however, it is not available in German language. The aim of this study was, therefore, to evaluate the reliability and validity of the German version of the OIDP in patients of a private practice.

**Material and methods:**

Translation of the original OIDP version was performed by a forward–backward process. Reliability was evaluated in terms of construct stability (test–retest) for the single items and the sum scores. The responsiveness to change in oral health status was assessed by pre- and post-treatment comparison, in addition. Validity was assessed as convergent validity in comparison with other dPROMs (OHIP-14; GOHAI) and objective dental findings.

**Results:**

A total of 330 patients participated in this study (mean age: 42.0 (18.0)). The OHRQoL of the participants was relatively high (OIDP score 4.3 (SD 14.3), OHIP score 4.8 (SD 5.3), GOHAI score 54.2 (SD 5.4)). A moderate construct stability for the total OIDP-score (ICC 0.686) was found whilst reliability for the single items varied between 0.179 (social contact) to 0.559 (showing teeth). Significant correlations were found for OIDP and OHIP (*p* < 0.001; *r* = 0.361) and OIDP and GOHAI (*p* < 0.001; *r* =  − 0.391) indicating moderate validity with a tendency to even stronger correlations for OIDP-s and OIDP-f (*r* ≥ 0.500).

**Conclusions:**

The German version of the OIDP demonstrated sufficient reliability and validity. OIDP’s general performance should be interpreted cautiously as the outcome was detected in a specifically healthy population.

**Clinical relevance:**

The OIDP is yet the only dPROM that evaluates both severity as well as frequency which makes validation interesting regarding specific target populations.

## Introduction

According to the World Health Organization’s (WHO) definition health is not only seen as pure absence of diseases rather than absolute physical, mental, and social well-being [1–4]. Measuring quality of life accounts for this postulation as subjective patient-reported outcomes (PROs) are evaluated [5]. Regarding medical issues, health-related quality of life (HQoL) is a subset of quality of life, and specific questionnaires are available; in turn, there are also dental patient-reported outcome measures (dPROM) to be used for dental patient-reported outcomes (dPRO) or oral health–related quality of life (OHRQoL) in particular. The construct of OHQRoL is based on patients’ impacts of oral problems, i.e., pain and functional impairment, but also includes social and psychological limitations. In addition, OHRQoL is modified by cultural surroundings and expectations with respect to dental care and care experiences [6–10]. DPROMs can be used—amongst epidemiologic applications and evaluations—i.e., for the process control during dental treatments. The most frequently used dPROM is the Oral Health Impact Profile (OHIP-49) and its derivates including population-adapted (i.e., children) and shorter versions [9, 10]. Using OHRQoL is administered by determination of impacts during the past month on a 5-point Likert scale. As an alternative, the Geriatric Oral Health Assessment Index is widely used in elderly populations as it merged into less bottom effects and only includes 12 items [6, 7]. However, most OHRQoL inventories solely access the presence of impacts. Their frequencies and severities are not evaluated separately. An exception is the Oral Impacts on Daily Performances (OIDP) [11]. Here, both frequency and severity are administered to build a sum score based on a multiplication. In, addition, the response burden is quite low as it only includes ten items. As the division of problems’ frequencies and severities has been seen as valuable in the evaluation of OHRQoL, OIDP was translated and validated in several languages [12–17]. However, to date, there is no German version of the OIDP. Thus, the aim of this study was to prove the reliability (construct stability and response to treatment) and validity (validated dPROM, objective dental findings) of a German version of OIDP in a cohort of German adults. The study hypothesis was that the German version of the OIDP is a reliable dPROM for the evaluation of OHRQoL.

## Materials and methods

### Protocol assignment and setting

Prior to the study start, the study protocol was handed to the local ethics committee of the University of Heidelberg and was approved under the number S-355/2017. Participants attending one private practice in the city of Heidelberg were recruited for this study. Inclusion criteria were solely being 18 years or older and having full legal capacity. All participants received written and oral information about the study background and the study procedures. Three-hundred and thirty-three patients gave informed written and oral consent and were included in the study.

### Translation process into German

**Fig. 1:**
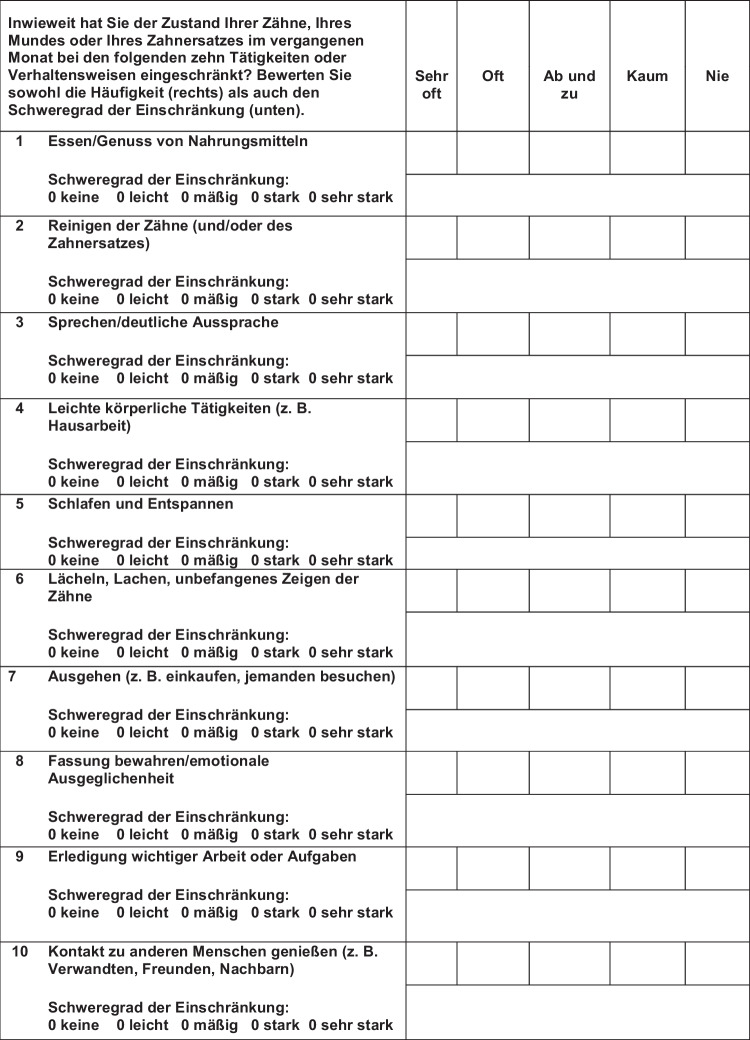
German version of the OIDP

A forward translation was performed by a bilingual professional translator. To verify its accuracy, the translation was scrutinized by two dentists for changes in the meaning of dental terminology. In case needed, the dentists were required to find a consensus concerning the correct meaning. The OIDP was then back-translated into English by another bilingual professional translator. All three versions (the original English version, the German version, and the back-translated version) were compared with each other and scrutinized for any differences or changes in meaning. If there were differences, both the professional translators and the dentists reviewed the respective parts and came to a consensus on the correct translation. This approach enabled the authors to preserve the proven validity of the original English tool (see Fig. 1 for the German version of the OIDP). This translation process is recommended to preserve validity in the literature [18, 19].

### Study procedures

Participants were asked to fill out the questionnaires. The reason for the dental consultation was administered (dental examination, acute pain, restorative treatment scheduled). In case of a dental examination appointment only, participants were asked to complete the questions 1 month later, in addition; in case of dental treatment or pain, participants were asked to complete the questionnaires a second time 1 month after the completion of the treatment. Questionnaires were sent to the participants via mail accompanied by a prepaid envelope.

### Collection of target determinants

Socio-demographic variables such as age, gender, and education were collected in a case record form, viz., standardized study sheets (CRF). One dentist (D. P.) complimented dental findings (number of teeth, dental status). The questionnaires also included a self-estimation of oral esthetics and function as well as global (oral) health (each dichotomized 0 = rather unhappy; 1 = rather happy).

### Oral health–related quality of life measures

OHRQoL was assessed by means of the Oral Health Impact Profile short version (OHIP-14) [9] and the Geriatric Oral Health Assessment Index (GOHAI) [6]. Furthermore, the German version of the OIDP was assessed. The OIDP comprises ten items which cover the dimensions pain/discomfort, psychosocial, and physical function. Each item has to be rated twice on a 5-point Likert scale ranging from 0, never, to 4, always, for the frequency and the severity of problems. The sum of all items, therefore, could give a value between 0 and 4 for frequency and severity rating as well as for the multiplication of frequency and severity (0–16). Thus, high scores of up to 160 can occur, indicating worse OHRQoL [11]. OHIP-14 scored in the same direction (low scores are indicative of good OHRQoL); GOHAI had a reciprocal scoring whereas high scores are indicative of good OHRQoL.

### Data processing and statistics

Completed CRFs were monitored by one dentist (D. P.) in the private practice and were entered into a database. A co-worker checked data on plausibility and legibility (B. P.) were monitored by a member of the University of Heidelberg (A.Z.).

Socio-demographic data and dental findings were computed by use of means (standard deviations, SD) and numbers (frequencies) according to data type. OIDP’s reliability was studied by construct stability (comparison of the two questionnaires without change in oral health) and response to treatment (second questionnaire after completion of treatment) along OIDP. Single items (frequency and severity separately as well as the multiplicated score) were evaluated by use of Kendall’s tau. Total sum scores were evaluated by use of intra-class correlations (ICC). In terms of validity, OIDP sum scores were correlated to GOHAI, OHIP-14, and objective dental findings by use of Pearson correlations. Pearson and Kendall’s correlations were interpreted as follows: 0.1–0.3, weak correlation; 0.3–0.5, moderate correlation; > 0.5, strong correlation. ICCs were interpreted as < 0.5, poor; 0.5–0.75, moderate; 0.75–0.9, good; and > 0.9, excellent. Positive correlations are in addition expressive for good homogeneity of items [20–22].

Statistical analysis was performed by means of SPSS Version 25. The level of local statistical significance was defined as *p* < 0.05.

## Results

### Study population

The mean (SD) age of the study population (*n* = 330) was 42.0 (18.0) years and slightly more than the half (58.2%) were female. The majority of participants (54.8%) had a university degree. The mean number of teeth present was 26.7 (SD 5.1) and out of all participants, 253 (76.7%) had natural teeth or fixed dental prostheses (partial dental prostheses: 74 (22.4%). The majority of the participants were rather happy with their oral esthetics (86.1%), oral function (94.2%), oral health (82.7%), and global health (92.7%).

The mean total OIDP score for all participants was 4.3 (SD 14.3, range 0–143), the mean OHIP score was 4.8 (SD 5.3, range: 0–39), and the mean GOHAI score was 54.2 (SD 5.4, range 14–60). Detailed characteristics are presented in Table 1.

**Table 1:** Participant characteristics (*n* = 330)

	Mean (SD)/number (percentage)	Range
Age	42.0 (18)	18–89
Gender		
Male	138 (41.8%)	-
Female	192 (58.2%)	-
Last dental visit (month)	9.4 (13)	0–130
Reason for a dental visit		
Dental examination	217 (66.0%)	-
Acute pain	29 (8.8%)	-
Restorative treatment scheduled	83 (25.2%)	-
Evaluation of oral esthetics		
Rather happy	284 (86.1%)	-
Rather unhappy	46 (13.9%)	-
Evaluation of oral function		
Rather happy	311 (94.2%)	-
Rather unhappy	19 (5.8%)	-
Evaluation of oral health		
Rather happy	273 (82.7%)	-
Rather unhappy	57 (17.3%)	-
Evaluation of global health		
Rather happy	306 (92.7%)	-
Rather unhappy	24 (7.3%)	-
Education		
Secondary school	17 (5.2%)	-
Secondary high school	64 (19.4%)	-
High school diploma	68 (20.6%)	-
College degree	38 (11.5%)	-
University degree	143 (43.3%)	-
OIDP	4.3 (14.3)	0–143
OIDP-s	2.4 (4.5)	0–39
OIDP-f	2.2 (4.4)	0–38
OHIP	4.8 (5.3)	0–39
GOHAI	54.2 (5.4)	14–60

### Reliability/construct stability

In participants without changes of oral health as measured by objective dental findings, Kendall’s tau analysis detected a moderate construct stability for nearly all items of the OIDP as well as moderate construct stability for the total OIDP-score (ICC 0.686) evaluated by use of intra-class correlations. In this context “cleaning teeth” was the worse (*τ* = 0.122, weak correlation), and “showing teeth” (*τ* = 0.559, strong correlation) was the most reliable item. Similar results were found for the solely evaluated OIDP-f and OIDP-s score (see Table 2). In participants with changes of objective oral health status/following dental treatment, correlations between items’ pre and post-scores as well as between the sum scores were ubiquitously not significant (see Table 3).

**Table 2:** Construct stability of the OIDP of participants without changes/dental treatment in oral health after 1 month (*n* = 55)

Item	C	*p*-value
Total OIDP score		
Eating	0.217	0.083
Cleaning teeth	0.112	0.324
Speaking	0.321	**0.017**
Physical activities	0.336	**0.013**
Sleeping/relaxing	0.483	** < 0.001**
Showing teeth	0.559	** < 0.001**
Going out	0.315	**0.019**
Emotional status	0.307	**0.018**
Carrying out work	0.324	**0.015**
Enjoy social contact	0.179	0.173
Total OIDP scores	ICC 0.686 (0.463–0.817)	
	*p* < 0.001	
**OIDP-frequency**		
Eating	0.214	0.089
Cleaning teeth	0.110	0.379
Speaking	0.284	**0.033**
Physical activities	0.330	**0.014**
Sleeping/relaxing	0.531	** < 0.001**
Showing teeth	0.494	** < 0.001**
Going out	0.265	**0.048**
Emotional status	0.271	**0.038**
Carrying out work	0.322	**0.016**
Enjoy social contact	0.180	0.173
Total OIDP-f score	ICC 0.571 (0.262–0.750)	
	*p* = 0.001	
**OIDP-severity**		
Eating	0.233	0.068
Cleaning teeth	0.104	0.416
Speaking	0.294	**0.029**
Physical activities	0.330	**0.014**
Sleeping/relaxing	0.496	** < 0.001**
Showing teeth	0.558	** < 0.001**
Going out	0.315	**0.019**
Emotional status	0.302	**0.021**
Carrying out work	0.340	**0.012**
Enjoy social contact	0.162	0.220
Total OIDP-s score	ICC 0.660 (0.418–0.802)	
	*p* < 0.001	

Significant *p*-values are marked in bold. *C*, correlations coefficient; single items (frequency and severity separately as well as the multiplicated score) were evaluated by use of Kendall’s tau. Total sum scores were evaluated by use of intra-class correlations

**Table 3:** Construct stability of the OIDP in participants with changes in oral health after 1 month (*n* = 79)

Variable	Coefficient	95% CI LB	95% CI UB	*p*-value
OIDP-f score	− 0.035	− 0.623	0.339	0.560
OIDP-s score	− 0.007	− 0.582	0.358	0.513
Total OIDP score	0.045	− 0.494	0.391	0.419

### Validity

**Fig. 2:**
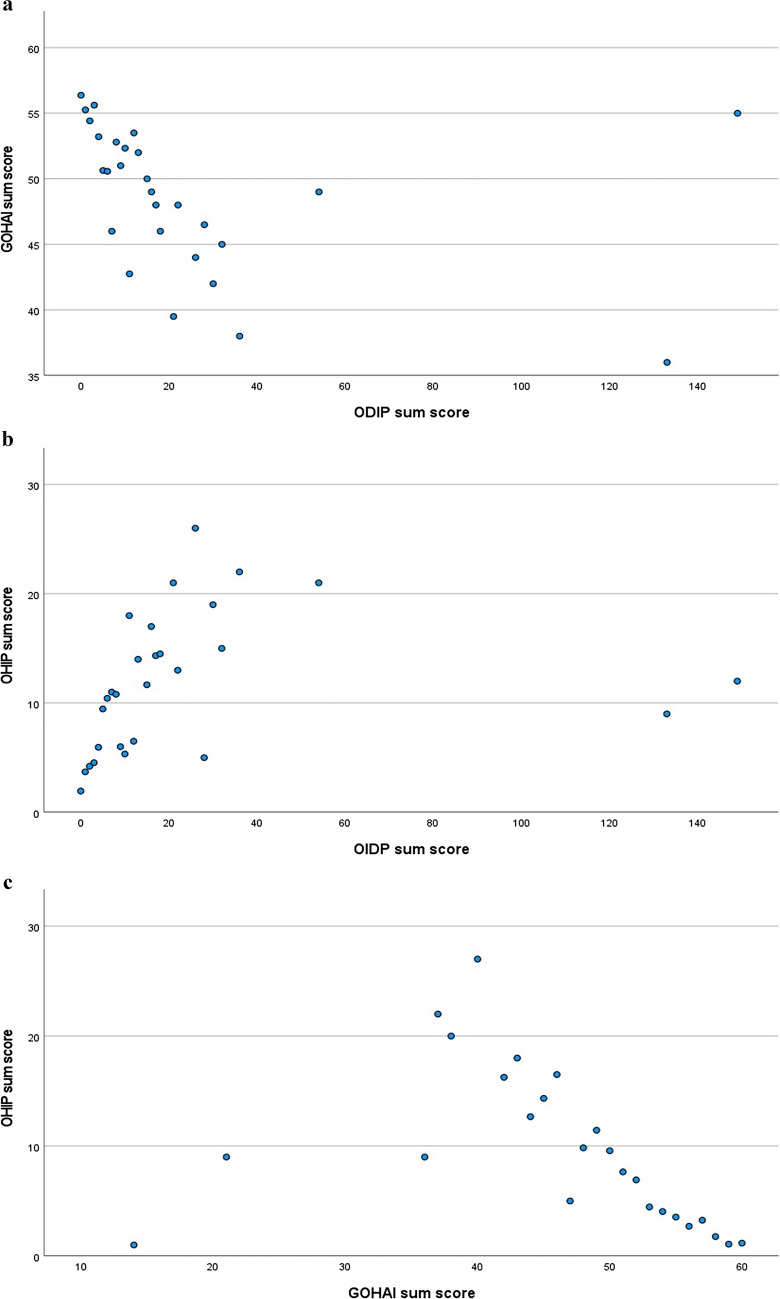
Correlations between the different dPROMs: **a** GOHAI vs. ODIP; **b** OHIP vs. OIDP; **c** GOHAI vs. OHIP

Pearson correlation detected significant correlations for OIDP and OHIP (*p* < 0.001; *r* = 0.361) and OIDP and GOHAI (*p* < 0.001; *r* =  − 0.391) (see Fig. 2). Correlations between OIDP-f and OIDP-s to OHIP were *r* = 0.527 and *r* = 0.546, respectively. OIDP-f and OIDP-s showed similar effect sizes to GOHAI, because of scaling direction with negative omens (*r* =  − 0.503; *r* =  − 0.500), indicative of moderate to strong validity. In terms of comparison, OHIP and GOHAI correlated *r* =  − 0.611. Furthermore change in oral health (*p* = 0.018), the reason for dental visit (*p* = 0.039), and evaluation of oral esthetic (*p* < 0.001) seem to be correlated with OIDP sum scores indicating the questionnaire’s validity. However, no significant correlations were found between, e.g., number of teeth, prosthetic status, or dichotomous self-estimation of oral function (*p* > 0.05) (see Table 4).

**Table 4:** Pearson correlation for the different variables and OIDP as the dependent variable for measurement of validity (*n* = 330)

*Variable*	*C*	*p*
Change in oral health	− 0.204	**0.018**
Reason for a dental visit	0.114	**0.039**
Evaluation of oral esthetics	0.181	** < 0.001**
Evaluation of oral function	0.049	0.374
Last dental visit	− 0.091	0.098
Number of teeth	0.022	0.687
Gender	0.074	0.178
OHIP	0.361	** < 0.001**
GOHAI	−0.391	** < 0.001**

all significant *p* values are marked in bold

## Discussion

The results of the study suggest that the German version of the OIDP is a predominately reliable and valid dPROM for the evaluation of the oral health–related quality of life of adults; thus, in an overall view, the study hypothesis had to be accepted.

When evaluating OIDP reliability which focused on the reproducibility of the values of items when the same subjects responded to the questionnaire twice, the correlation coefficients was acceptable. All the inter-item correlations were positive, thus demonstrating acceptable internal consistency and acceptable homogeneity of the items. This result is similar to the results of the Malaysian version of the OIDP [23]. With respect to reliability, the ICC higher than 0.60 found in this study was comparable to other international adaptions of the OIDP and indicative of acceptable construct stability of the German version of the OIDP [12–17]. When testing validity, the German version of the OIDP was in acceptable agreement with other measures of self-perceived OHRQoL. However, the OIDP has similar mean scores and a higher standard deviation compared to the OHIP which is maybe indicative of partially minor bottom effects in a rather healthy study cohort. In addition, the OIDP might be more specific in a study population with more frequent and greater impairments. One could further speculate that the division of the OIDP in the query of severity and frequency might lead to an increased sensitivity of the questions due to the multiplication of the two sub-scores per item, i.e., seldom burden (1 point in OIDP-f) combined with very strong severity (5 points in OIDP-s) or vice versa. This could be another reason for the rather high standard deviation. This assumption is supported by the fact that OIDP-s and OIDP-f in separate view showed standard deviations comparable to that of OHIP whilst the mean score is even smaller. Nevertheless, the OIDP is the only screening assessment tool that evaluates severity and frequency and has therefore a justification and importance if a more dedicated OHRQoL is required (insight into frequency and severity). When interpreting the results, it should be kept in mind that the different dPROMs were validated in different study populations. For example, the GOHAI was developed for the elderly [6, 7]. It is not surprising, that this community usually has different impairments compared to young and healthy persons which were investigated in this study setting. The focus of the different dPROMs is therefore shifted. This could explain the weaker correlation between OHIP and GOHAI in this study population. Furthermore, it has to be kept in mind that Hassel et al. found similar correlations in their validation study (GOHAI vs. OHIP: − 0.721) [6]. It further should be kept in mind that the response to treatment reliability was rather low, presuming that the impact of treatment on the patients was very inhomogenous from less invasive to very extensive.

Another interesting result is that this study found no association between OIDP scores and gender, age group, or level of education. These results are in accordance with Ostberg et al., in Swedish, and Razanamihaja and Ranivoharilanto et al. in the Malaysian version [12, 17].

Although the sample size was not calculated prior to the study because it is explorative in nature, the sample size was much larger than recommended in the literature [23, 24] and therefore sufficient for testing reliability and validity. The responsiveness to treatment and construct stability, however, was tested in a small number of patients only, but this group was inhomogeneous as all patients received different treatment under comparable treatment conditions. However, the result suggests that the German OIDP responds to changes in clinical factors, as expected. In this context, it must be noted the prevalence of negative impact on oral health was low in this study population. This can be again explained by a young and healthy study population and a high number of natural teeth present (mean 26.1) as well as many fixed dental prostheses rather than removable dentures. Those factors are described to have a high impact on OHRQoL [25–27] and 94.2% of the study population stated that they were happy with their oral function. For participants with natural teeth and fixed dental prosthesis, individual problems like tooth color or tooth position might be more relevant than the chewing function.

### Strength and weaknesses

This study is the first study that evaluated the German version of the OIDP. The translation of the original version did not come up against major difficulties as there are only minor cultural differences. Furthermore, a forward-backword translation process was used which is recommended to preserve validity in the literature [18, 19]. Another strength of the study is that in contrast with other validation studies, the clinical data were not solely self-reported. It was recorded after examination by a dentist. Limitations are the relatively young and healthy study population and that construct stability/response to treatment was only tested in a rather small population.

## Conclusion

The OIDP is the only validated dPROM yet that evaluates both severity and frequency and is therefore of high interest. The German version of the OIDP seems to be predominately reliable and valid for the administration of OHRQoL. However, results may not unreservedly be extrapolated to other populations.

## Data Availability

The datasets used and/or analysed during the current study are available from the corresponding author on reasonable request.
